# Ecraseur

**Published:** 1858-11

**Authors:** 


ECRASEUR.—Dr. Frederick D. Lente of Cold Spring, New York, in a letter to the senior editor, writes in the following terms of the Ecraseur, as used on the continent:—

"I spent my time entirely in Paris, London, and Dublin, mostly in Paris, where I saw many interesting surgical operations, and some novelties. I was particularly pleased with the operation of the Ecraseur in the hands of its inventor, M. Chassaignac. To appreciate its full value, and extensive range of application, one must see him use it, which he certainly does with extraordinary tact and success. Among the operations which I witnessed, I may mention amputation of the *cervix uteri.* He merely dissects the cervix a little from its connection with the bladder, which he considers important, and transfixes it with a trocar to prevent the chain from slipping before applying the latter. In all cases, especially when the part is very vascular, he proceeds *very* slowly to work the lever, resting some seconds between each click of the instrument. This he considers absolutely necessary to the full success of the operation as regards hemorrhage. Another operation was the removal of a large cancerous tumor of the *rectum*, involving the verge of the anus. In order to get the chain completely around and above the base of the tumor, he first transfixed its base from behind forward with a trocar, the point coming out at the perineum; he next passed the chain through the canula of the trocar, which he then removed, and proceeded to divide the tumor into two halves. He then, successively, and without difficulty, amputated each half, first passing a trocar through the base to prevent the chain from slipping from its place. The hemorrhage was trifling compared with what generally occurs in these operations with the knife, and required only the application of a compress and T-bandage.

"He also operated for *varicocele.* First, isolating the veins of the cord from its other constituents, he passed three large needles under them at a little distance from each other, then throwing the chain around them, and thus inclosing a loop of the veins and a portion of the integument of the *scrotum;* this was soon removed by working the lever, without so much as staining the skin with blood. Maisonneuve at La Pitié has, as you are aware, been for some months past amputating limbs with his Ecraseur, which works with a screw instead of a double acting lever. His idea is that he will thus incur less hazard of purulent absorption, which has been carrying off a large majority of his victims of amputation. Dr. Luckley describes his method in a recent number of the *N. Y. Journ. of Medicine.* He argues from the observed fact that comparatively few cases of compound fracture die of this and other allied affections. So he makes his amputations as similar to a case of compound fracture as possible. I saw one stump of a leg entirely healed, that had been subjected to this, in appearance, barbarous procedure, and was informed by American physicians in Paris, who had watched the progress of his cases, some nine or ten I think, that had undergone amputation by this method, that the mortality was decidedly reduced.

"Whether it is a case of *propter hoc* remains, I think, to be decided by a greater number of cases, and a greater lapse of time. Maisonneuve has also been using injections of the perchloride of iron quite extensively, and with good success, for the obliteration of varicose veins. The strength of the liquid used by him is 30° of Baumé, or about one-third of the solid salt, which is too deliquescent to be long kept in this form, to two-thirds of water. A somewhat weaker solution he also uses with success, to arrest hemorrhage from small arteries, and profuse oozing of blood during bloody operations. I know that this agent has also been used in this country for the above objects, but not very extensively."

